# Economic analyses of behavioral health intervention implementation: Perspective on stakeholder engagement

**DOI:** 10.3389/fpsyt.2022.1031325

**Published:** 2022-12-21

**Authors:** Rebecca A. Raciborski, Eva N. Woodward, Jacob T. Painter

**Affiliations:** ^1^VA Center for Mental Healthcare and Outcomes Research, Central Arkansas Veterans Healthcare System, North Little Rock, AR, United States; ^2^Evidence, Policy, and Implementation Center, Central Arkansas Veterans Healthcare System, North Little Rock, AR, United States; ^3^Department of Psychiatry, College of Medicine, University of Arkansas for Medical Sciences, Little Rock, AR, United States; ^4^Division of Pharmaceutical Evaluation and Policy, College of Pharmacy, University of Arkansas for Medical Sciences, Little Rock, AR, United States

**Keywords:** behavioral health, implementation, cost, economic analysis, cost-effectiveness, budget impact, stakeholder engagement, economic evaluation

## Abstract

To provide full potential benefits to patients, behavioral health interventions often require comprehensive and systematic implementation efforts. The costs of these efforts should therefore be included when organizations decide to fund or adopt a new intervention. However, existing guidelines for conducting economic analyses like cost-effectiveness analyses and budget impact analyses are not well-suited to the complexity of the behavioral healthcare pathway and its many stakeholders. Stakeholder engagement, when used effectively with recent innovations in economic analysis, advance more equitable access to interventions for individuals living with behavioral health conditions. But early and ongoing stakeholder engagement has not yet been incorporated into best-practice guidelines for economic evaluation. We discuss our perspective, as researchers and clinicians in a large integrated health system, on how the integration of stakeholder engagement with existing economic analysis methods could improve decision-making about implementation of behavioral health interventions.

## Introduction

Treating behavioral health conditions is imperative. Mental health and substance disorders are the world’s leading cause of disability and fifth highest cause of death (1). Treatment is also expensive; treatment of major depressive disorder in the U.S. alone exceeds $300 billion annually (2). If a new efficacious intervention is introduced, how much benefit will it provide in improved function or reduced mortality? What will it cost to implement it with fidelity so that it is effective in practice? Is it affordable? Economic analyses aim to answer these kinds of questions. Like clinical trials, they are conducted with the goal of obtaining sufficient information to make a policy decision. These analyses are a standard method by which policymakers and payers decide if a new treatment should be available. However, existing pharmacologic-focused guidelines for conducting economic analyses do not easily extend to behavioral health interventions (2, 3) or their implementation (4).

While there are myriad challenges, many relate to the complexity of the behavioral healthcare pathway. Costs and benefits of behavioral health interventions are distributed unevenly and in meaningful ways to stakeholders beyond the payer and patient. When successful treatment depends on patients, their close contacts, and their clinicians, then decision makers are best served by analyses that incorporate these other perspectives. We believe that engaging all stakeholders increases the usefulness of economic analyses as a decision-making tool. We discuss our perspective on the challenges of applying economic analysis to behavioral health interventions, implementation thereof, and innovations in the field that may potentially improve equity in economic analyses.

## Economic view of implementing behavioral health interventions

Implementing behavioral health interventions requires significant investment; trainings and manualized protocols for psychotherapies are not sufficient to ensure psychotherapies are used by clinicians (5). Other strategies are required to implement behavioral healthcare interventions, increase adoption by providers, and reach more patients. Such implementation strategies may include changing infrastructure (e.g., physical space alterations and re-organizing teams), increasing demand among patients through marketing, and engaging relevant personnel at multiple levels (e.g., leadership and frontline staff) (6). The economist sees a cost attached to not only new space or printed marketing materials, but the time spent training teams on new workflow and meetings to create buy-in.

When deciding to implement an intervention, economic analysis is one approach to inform organizational decision-making. In our work, economic analyses inform decisions about providing and funding behavioral health interventions in the US Department of Veterans Affairs (VA). Cost-effectiveness analyses have long been a feature of VA clinical trial research (7, 8); and research about implementation of new behavioral health interventions is incorporating these analyses too. VA’s Quality Enhancement Research Initiative now requires a budget impact analysis before implementing new interventions. We focus in this article on two methods we use most frequently as they most often meet needs of payers and decision makers: cost-effectiveness and budget impact analyses. Table 1 provides a brief overview of the two approaches. Other approaches (e.g., cost benefit analysis) also have applications to psychiatric care. Luyten et al. (9) provide an introduction and Knapp and Wong (10) provide a comprehensive review from a psychiatric perspective.

**TABLE 1:** Comparison of cost effectiveness and budget impact analysis.

	Cost effectiveness analysis	Budget impact analysis
Decision informed	Is the intervention worthwhile?	Can the intervention be afforded within current budget constraints?
Typical audience	Policymakers	Budget holders within an organization
Outcome of interest	Disease/condition-specific clinical endpoint *or* generic utility-based measure for quality of life	Changes in treatment mix/resource use after introduction of new intervention
Method of assigning value to benefits	Direct (or “natural”) measurement of clinical endpoints *or* socially determined preference-weights for quality-of-life measures	Currency-denominated accounting cost that is avoided (if any)
Costs included	Health sector perspective: Formal health sector costs Additional cost for societal perspective: Informal caregiver costs, Non-health sector costs, Productivity losses	Costs incurred by the payer (typically only health sector costs are incurred)
Method of assigning value to costs	Currency-denominated economic cost, including value of opportunity cost	Currency-denominated accounting cost

Cost-effectiveness analysis and budget impact analysis have different analytic goals but share some features. Cost-effectiveness analyses classically support decisions about whether an intervention should be made available to patients or to which patients it will be made available if there are heterogeneous treatment effects. Recently, they have examined the relative value of competing implementation strategy bundles to enhance uptake of behavioral health interventions (11, 12). By contrast, budget impact analyses support decisions about whether an intervention can be made available given the payer’s budget or under what conditions it would be possible to do so (e.g., patient copayment). The “payer” is typically an insurer or national health service. When evaluating implementations, the payer is more likely to be the adopting organization alone because a new intervention’s implementation is usually not directly reimbursable.

Care for behavioral health conditions is complex with many people involved in its delivery and use. An intervention incurs cost at each stage of the behavioral healthcare pathway. Some costs are easily identifiable (e.g., amount an insurer pays for a counseling session). But others are more complex. An office visit minimally involves scheduling clerks, screening technicians, and provider teams; inpatient and emergent care requires an even more diverse mix of staff and material resources. The immediate cost of time, measured by each staff member’s wage and fringe benefit rate, and other operating expenses is borne by the organization providing care. Ideally, insurer payments are sufficient to compensate for these costs. Less common “costs” to providers are more qualitative in nature (e.g., managing higher severity conditions). Receiving care also requires time from patients and, in the case of conditions involving diminished capacity (e.g., severe posttraumatic stress disorder), their caregivers.

Finally, there are costs associated with the condition the intervention seeks to ameliorate. For payers, these costs are incurred because of disease complications (e.g., psychiatric hospitalization). For providers, cost may be increased time for patient disease management and care coordination or the added stress of caring for a patient in crisis. The greatest costs though tend to be borne by patients (e.g., lost wages when unable to work) and their informal caregivers (e.g., spouse’s uncompensated time). Condition-related costs also include reduced quality of life for patients and, in the case of severe conditions (e.g., schizophrenia), for their families and close contacts. Some conditions reach even further into society (e.g., through cost of supportive housing).

Weighed against costs are the tangible and intangible benefits of treatment to patients, their families, close contacts, and the societies in which they live (e.g., improved health, jobs retained, and relationships stabilized). Providers benefit from seeing patient improvements and from implementation of interventions that improve workflow and reduce stress. Healthcare organizations benefit when implementation strategies are selected for cost-effectiveness and increased confidence in decisions. Payers benefit by avoiding the cost of disease complications.

The approach to measuring costs and benefits differs based on analytic goals. Cost-effectiveness analysis quantifies the benefit as a relative gain in some measure of health improvement (e.g., hospitalizations avoided) compared to the cost to health system or society. When benefits are measured in years added to a patient’s life and weighted for the quality of life experienced in those additional years, i.e., *quality-adjusted life years* (QALYs), analyses may be called *cost-utility analyses*. Following others (13), we include analyses with benefits measured in QALYs in our use of “cost-effectiveness analysis.” By contrast, budget impact analysis includes only cost incurred by the payer; benefits enter only if the payer avoids a cost. The two analyses differ in the time frame over which the measurement occurs as well. Cost-effectiveness analyses tend to be long-run projections while budget impact analyses are usually confined to a 1-to-5-year period based on the payer’s budgetary planning cycle.

Regardless of method, the analytic team makes choices about what costs and benefits are relevant to the decision and how to measure them (e.g., where the data comes from, how detailed it should be, over what period it is gathered). These choices rely on assumptions about importance and magnitude. The assumptions set and calculations it leads to are referred to as the economic *model structure*. For both cost-effectiveness and budget impact analyses, the degree of uncertainty about costs and benefits increases the farther into the future projections occur. When this uncertainty is due to decisions about the assumptions themselves, it is called *structural uncertainty*.

## Challenges analyzing behavioral health interventions and their implementation

The complexity of behavioral healthcare makes fundamental decisions that determine model structure particularly challenging. Challenges begin with defining which patients should be considered “treated” and what the intervention costs. Behavioral interventions like psychotherapy are tailored to patient needs; completion of treatment and likelihood of obtaining full benefits varies by patient. The amount of time, and thus labor, also depends on individual patient needs. Labor costs vary by provider type, geographic region, and other local factors. This contrasts with pharmaceuticals, which generally have a common “list price” from the payer’s perspective.

Further, while efficacy and patient adherence to treatment mostly determine outcomes from pharmaceutical treatment, effectiveness of behavioral health interventions also depends on clinician fidelity. Measures of fidelity are sometimes gathered in the context of a trial but rarely collected otherwise. At the clinic or organizational level, proper implementation is necessary to ensure fidelity and thus patient improvements. The cost of the implementation effort is thus relevant to the payer but the implementation strategies, like the intervention itself, lack a list price (4, 14). Implementation cost data may be collected during a trial using existing methods (15) but they likely overstate cost in practice when cost per patient declines as each clinician treats more patients and as caseloads increase beyond typical trial size (3).

Another complication is the relative heterogeneity of the “usual care” comparator across geographic regions, healthcare systems, and individual providers. Standard treatment of some behavioral health conditions has been codified. But for many, a variety factors contribute to what is considered usual care, such as duration of sessions in scheduling grids, match between patient severity and available services, or local norms regarding therapeutic orientation.

In cost-effectiveness analysis, these challenges are compounded when determining relevant costs and benefits. Recommendations are clear that an enhanced health-system perspective, and ideally a societal perspective, should provide the reference case (13). When non-health system costs and benefits are high, the choice between the health system and societal perspective can alter the conclusion about whether an intervention is cost-effective (16, 17). Regardless, it is common for analyses to omit costs and benefits that are time consuming to gather or that researchers do not conceptualize as relevant (17). Yet, many behavioral health interventions result in substantial non-health system costs (e.g., caregiver time) and benefits (e.g., opioid use disorder therapy reducing criminal justice involvement) (3, 10). Behavioral health interventions are subject to substantial uncompensated patient time costs (3), such as time spent integrating practices learned in psychotherapy into daily life. Neglecting these costs could skew the estimated cost-effectiveness if they ultimately change behavior of non-payer participants in the behavioral healthcare pathway.

Budget impact analysis confronts a similar challenge, even though defining costs from the payer perspective seems straightforward (18). The payer perspective may oversimplify how organizational change occurs, particularly for implementation of new behavioral health interventions with multiple intra-organization budget holders and stakeholders who incur disproportional costs (e.g., an integrated health system implements emergency department-based suicide screening that refers more patients to specialty mental health). Additionally, outside of integrated systems, budget impact analyses conducted from an insurer’s perspective omit implementation costs incurred by providers (e.g., time spent training for required credentials) unless these costs are reimbursed.

Striking the right balance of detail for all stakeholders is critical. If the analytic team spends time gathering data on costs that ultimately have little effect on the conclusion, the timeliness of the analysis to the decision maker is reduced.

## Discussion

Understanding needs of decision-makers and matching these to the knowledge of other stakeholders ensures the analysis provides useful information. Importantly, our operational and clinical partners often have concerns beyond cost, including health equity, when considering implementing an intervention. Understanding their concerns and successfully using other stakeholders’ knowledge requires early and frequent engagement. Stakeholder engagement spans the continuum of intensity from unidirectional consultations (least intensive) to potentially co-produced evaluations (most intensive) (19).

As applied to economic analysis, stakeholder engagement can help specify relevant analytic goals. Applying principles of community-based participatory research (20) and implementation science (21) to engage those most affected by the decision being made may also make economic analyses more equitable. Stakeholder engagement can also help determine and refine the economic model structure when faced with the complexities of behavioral healthcare. Although economic analyses are increasingly acknowledging a role for stakeholder engagement, best-practice guidelines have not yet incorporated advice for when or how to do so.

### Specifying analytic goals

Identifying and communicating with relevant stakeholders is essential for economic analyses. An analysis that omits key components needed for the decision leaves decision makers no better informed, and possibly worse, if the wrong conclusion is presented. While cost is a necessary consideration for budget holders, it may need to be weighed against other stakeholder-identified factors. If so, stakeholders can be involved when developing an economic analysis plan to specify goals. Despite its promise, this practice is not widely adopted (22) and has mostly involved pharmaceutical manufacturers requesting input from regulatory agencies (23).

More recently, implementation scientists are focusing on cost as a factor that enables or hinders uptake of an intervention and the value of cost information to decision-makers (4, 24, 25). What staff and providers stand to gain from offering or improving behavioral health interventions depends on their context (e.g., funding structures, existing capacity for quality improvement). Depending on their role, they may incur substantially different costs as well, which can influence the rapidity of the new intervention’s diffusion in practice. Knowing such information in advance may help anticipate reluctance to engage during implementation.

### Scoping and measuring costs and benefits with stakeholder engagement

Decision makers and other stakeholders exist all along the behavioral healthcare pathway. It is essential to ensure that senior leadership and core staff involved in quality improvement at the organization are involved in scoping and measuring cost and benefits (21, 26). This improves decision maker confidence and minimizes the risk that the model structure relies on poor assumptions about costs and benefits (22). For example, when conducting a budget impact analysis, stakeholders within an organization can rely on institutional knowledge to highlight areas where structural uncertainty may exist, particularly in later years. Consider the case of rapid organic practice change. If a new behavioral intervention is being introduced when “usual care” patterns are already shifting, frontline staff will be best positioned to know this. Economists can then incorporate this knowledge with alternate scenarios to present more useful information.

As a technical matter, stakeholder engagement can reduce the analytic burden and ensure primary data collection efforts are focused where the value of information is highest. For example, in the absence of a standard list price for a behavioral health intervention, clinicians can identify the current local standard of practice and which components of usual care are most relevant. Clinical leadership can clarify which strategies and processes are required for implementation and how long it takes an organization to move from planning to offering a new intervention to patients. It is possible to quantify the value of additional information through a formal value of information analysis (27). Such analyses have become more common when conducting cost-effectiveness analyses.

Early and ongoing stakeholder engagement with those who will bear cost or stand to benefit from an intervention during implementation also helps identify if multiple perspectives are needed when scoping and measuring costs and benefits (25, 28, 29). Incorporating other stakeholder perspectives on costs and benefits offers insights to behaviors like treatment engagement, adherence, and clinician adoption of and fidelity to interventions. This may mean incorporating measures of departmental level cost within the organization to determine if cost is being shifted. Or, to the extent that provider-assessed value influences adoption of promising new interventions, it may mean adding non-cost outcome measures salient to providers (e.g., patient gains and reduced caseload). Because the benefits and costs do not always accrue to the same stakeholders, conflicts may arise. Many approaches to resolving such conflicts have been proposed (30–32) and their application to stakeholder engagement in economic analyses should be explored in future work.

### Equity in economic analyses of behavioral health interventions

We use the term “health equity” broadly, referring to a range of ethical concerns from opportunity to achieve full health to respect for individual autonomy (33). As a US federal agency, VA must now incorporate equity into its program evaluations (34). As more program evaluations incorporate an economic component, equity concerns and economic analyses are increasingly intersecting. Fortunately, several innovations in economic analysis methods, along with insights from other fields, support addressing these concerns.

Evaluators can incorporate equity into the economic analysis process as early as goal specification. For example, decision-makers may request having patient financial costs included alongside a traditional budget impact analysis if they want to identify which of similar interventions is the least burdensome to patients. The emerging field of distributional cost-effectiveness analysis provides a framework for explicitly considering questions about how an intervention changes the distribution of health, health service access, cost, and protection from financial risk (35). Approaches borrowed from other fields, such as community-based participatory research, further increase equitable practices when those affected by the decision are given a greater voice in how it is framed.

Scoping costs and benefits is another potential point of intersection between economic analysis and equity. Economists necessarily specify the initial model structure; their choices about what costs and benefits are important will be shaped by their lived experience. However, stakeholders along the full continuum of the behavioral healthcare pathway may prioritize costs and benefits differently from those scoped into economic analyses with the standard decision-maker perspective. Patients likely have different perceptions of value (36), especially those like individuals living with serious mental illness who experience marginalization or are part of minoritized populations. Teams can engage in reflexivity practices throughout the process by explicitly considering differences in perceived value to enhance a focus on ethical, equitable use of economic evaluation (37, 38). Frequent interaction should be continued until the economic analysis is complete, with the team asking questions, sharing updates, and listening to and incorporating stakeholder feedback (26).

Once the scope is determined, equity can also inform decisions about processes for measuring costs and benefits. For example, many US federal agencies prohibit the use of cost-per-QALY measures to make decisions about whether an intervention will be available to patients, though some permit comparisons to choose between treatments for the same cohort of patients (39). The primary factor leading to US restrictions on QALYs was concern about discriminatory effects for people with chronic health conditions (39). Adopting more sensitive measures of health status change and better assessment of the value of that change could mitigate potential discrimination. Organizations, like VA, that conduct trials and have access to large populations of patients with behavioral health conditions are ideally positioned to further develop instruments that capture changes in quality of life salient to individuals with behavioral health conditions (40–42) and explore how decisions change when of using quality of life valuations from those with experience of a behavioral health condition instead of standard societal valuations (43). Research should also explore the result of incorporating spillover quality of life effects in analyses of behavioral health interventions and their implementation (44).

Economic analysis and equity also intersect when the decision is made to implement an intervention. Distributional cost-effectiveness analysis provides economists with a framework for ensuring decision-makers have sufficient information to balance total health and equity tradeoffs. Another approach is the individualized comparative effectiveness framework that focuses on the relative benefit of different interventions for subgroups of patients (45). This information can be used by clinicians, patients, and caregivers to assume a greater role in the decision-making process.

As an example of where these principles apply, consider integration of mental health services into primary care, which reduces depression symptoms in patients and costly utilization (46, 47). A traditional economic analysis may be a cost-effectiveness analysis for policymakers considering endorsing the change or budget impact analysis for clinic owners. An equitable analysis would identify any pre-existing inequities that may be exacerbated. For example, decision-makers may want to know if the intervention disproportionately benefits patients who already have good access to primary care services. To be relevant to patients, the analysis also may include multifaceted “success” metrics that address symptom reduction and quality of life. Finally, integration may affect job satisfaction and burnout for primary care clinicians (48). Implementation scientists could use the results of such an analysis to design approaches to mitigate clinician burnout effects.

## Data availability statement

The original contributions presented in this study are included in the article/supplementary material, further inquiries can be directed to the corresponding author.

## Author contributions

RR wrote and edited the manuscript, conceptualized framing with respect to economic analysis challenges and new developments, and provided references to key literature. EW wrote and edited the manuscript, conceptualized framing in relation to clinical audience and in context of implementation, and provided references to key literature. JP wrote and edited the manuscript and provided references to key literature. All authors contributed to the article and approved the submitted version.

## References

[1] Whiteford H Ferrari A Degenhardt L Feigin V Vos T. The global burden of mental, neurological and substance use disorders: an analysis from the Global Burden of Disease Study 2010. *PLoS One.* (2015) 10:e0116820. 10.1371/journal.pone.0116820 PMC4320057 25658103

[2] Proudman D Greenberg P Nellesen D. The growing burden of major depressive disorders (MDD): implications for researchers and policy makers. *PharmacoEconomics.* (2021) 39:619–25. 10.1007/s40273-021-01040-7 34013439 PMC8134814

[3] Shearer J McCrone P Romeo R. Economic evaluation of mental health interventions: a guide to costing approaches. *Pharmacoeconomics.* (2016) 34:651–64. 10.1007/s40273-016-0390-3 26922076

[4] Wagner T Yoon J Jacobs J So A Kilbourne A Yu W Estimating costs of an implementation intervention. *Med Decis Making.* (2020) 40:959–67. 10.1177/0272989X20960455 33078681

[5] Duncan B Miller S. Treatment Manuals Do Not Improve Outcomes. In: Norcross JC Beutler LE Levant RF editors. *Evidence-based practices in mental health: Debate and dialogue on the fundamental questions.* Washington D.C: American Psychological Association Press (2005).

[6] Waltz T Powell B Matthieu M Damschroder L Chinman M Smith J Use of concept mapping to characterize relationships among implementation strategies and assess their feasibility and importance: results from the Expert Recommendations for Implementing Change (ERIC) study. *Implement Sci.* (2015) 10:1–8. 10.1186/s13012-015-0295-0 26249843 PMC4527340

[7] Liu C Hedrick S Chaney E Heagerty P Felker B Hasenberg N Cost-effectiveness of collaborative care for depression in a primary care veteran population. *Psychiatr Serv.* (2003) 54:698–704. 10.1176/appi.ps.54.5.698 12719501

[8] Pyne J Fortney J Tripathi S Maciejewski M Edlund M Williams D. Cost-effectiveness analysis of a rural telemedicine collaborative care intervention for depression. *Arch Gen Psychiatry.* (2010) 67:812–21. 10.1001/archgenpsychiatry.2010.82 20679589

[9] Luyten J Naci H Knapp M. Economic evaluation of mental health interventions: an introduction to cost-utility analysis. *Evid Based Ment Health.* (2016) 19:49–53. 10.1136/eb-2016-102354 27075444 PMC10699413

[10] Knapp M Wong G. Economics and mental health: the current scenario. *World Psychiatry.* (2020) 19:3–14. 10.1002/wps.20692 31922693 PMC6953559

[11] Eisman A Hutton D Prosser L Smith S Kilbourne A. Cost-effectiveness of the Adaptive Implementation of Effective Programs Trial (ADEPT): approaches to adopting implementation strategies. *Implement Sci.* (2020) 15:1–3. 10.1186/s13012-020-01069-w 33317593 PMC7734829

[12] Dopp A Hanson R Saunders B Dismuke C Moreland A. Community-based implementation of trauma-focused interventions for youth: Economic impact of the learning collaborative model. *Psychol Serv.* (2017) 14:57. 10.1037/ser0000131 28134556 PMC5327921

[13] Sanders G Neumann P Basu A Brock D Feeny D Krahn M Recommendations for conduct, methodological practices, and reporting of cost-effectiveness analyses: second panel on cost-effectiveness in health and medicine. *JAMA.* (2016) 316:1093–103. 10.1001/jama.2016.12195 27623463

[14] Eisman A Kilbourne A Dopp A Saldana L Eisenberg D. Economic evaluation in implementation science: Making the business case for implementation strategies. *Psychiatry Res.* (2020) 283:30752–8. 10.1016/j.psychres.2019.06.008 31202612 PMC6898762

[15] Cidav Z Mandell D Pyne J Beidas R Curran G Marcus S. A pragmatic method for costing implementation strategies using time-driven activity-based costing. *Implement Sci.* (2020) 15:28. 10.1186/s13012-020-00993-1 32370752 PMC7201568

[16] Sullivan S Mauskopf J Augustovski F Caro J Lee K Minchin M Budget impact analysis—principles of good practice: report of the ISPOR 2012 Budget Impact Analysis Good Practice II Task Force. *Value Health.* (2014) 17:5–14. 10.1016/j.jval.2013.08.2291 24438712

[17] Drost R van der Putten I Ruwaard D Evers S Paulus A. Conceptualizations of the societal perspective within economic evaluations: a systematic review. *Int J Technol Assess Health Care.* (2017) 33:251–60. 10.1017/S0266462317000526 28641592

[18] Fairley M Humphreys K Joyce V Bounthavong M Trafton J Combs A Cost-effectiveness of treatments for opioid use disorder. *JAMA Psychiatry.* (2021) 78:767–77. 10.1001/jamapsychiatry.2021.0247 33787832 PMC8014209

[19] Goodman M Sanders Thompson V. The science of stakeholder engagement in research: classification, implementation, and evaluation. *Transl Behav Med.* (2017) 7:486–91. 10.1007/s13142-017-0495-z 28397159 PMC5645283

[20] Wallerstein N Duran B. Community-Based participatory research contributions to intervention research: The intersection of science and practice to improve health equity. *Am J Public Health.* (2010) 100(Suppl 1):S40–6. 10.2105/AJPH.2009.184036 20147663 PMC2837458

[21] Kirchner J Kearney L Ritchie M Dollar K Swensen A Schohn M. Research & services partnerships: Lessons learned through a national partnership between clinical leaders and researchers. *Psychiatr Serv.* (2014) 65:577–9. 10.1176/appi.ps.201400054 24585229

[22] Xie R Malik E Linthicum M Bright J. Putting stakeholder engagement at the center of health economic modeling for health technology assessment in the United States. *Pharmacoeconomics.* (2021) 39:631–8. 10.1007/s40273-021-01036-3 33982198 PMC8166701

[23] Maignen F Osipenko L Gajraj E Chivers R. Trends in early engagement between industry and HTA: analysis of scientific advice service provided by Nice since 2009. *Value Health.* (2014) 17:A441. 10.1016/j.jval.2014.08.1159 27201182

[24] Saldana L Ritzwoller D Campbell M Block E. Using economic evaluations in implementation science to increase transparency in costs and outcomes for organizational decision-makers. *Implement Sci Commun.* (2022) 3:40. 10.1186/s43058-022-00295-1 35410434 PMC9004101

[25] Eisman A Quanbeck A Bounthavong M Panattoni L Glasgow R. Implementation science issues in understanding, collecting, and using cost estimates: a multi-stakeholder perspective. *Implement Sci.* (2021) 16:75. 10.1186/s13012-021-01143-x 34344411 PMC8330022

[26] Sharek P Mullican C Lavanderos A Palmer C Snow V Kmetik K Best practice implementation: lessons learned from 20 partnerships. *Jt Comm J Qual Patient Saf.* (2007) 33:16–26. 10.1016/S1553-7250(07)33120-6 18277636

[27] Sculpher M Basu A Kuntz K Meltzer D. Reflecting uncertainty in cost-effectiveness analysis. Second ed. In: Neumann P Sanders G, Russell L Siegel J Ganiats T editors. *Cost Effectiveness in Health and Medicine.* Oxford: Oxford University Press (2017). p. 289–318. 10.1093/acprof:oso/9780190492939.003.0011

[28] Delafield R Hermosura A Ing C Hughes C Palakiko D Dillard A A community-based participatory research guided model for dissemination of evidence-based interventions. *Prog Commun Health Partnersh.* (2016) 10:585. 10.1353/cpr.2016.0067 28569684 PMC5458619

[29] Bauer M Miller C Kim B Lew R Weaver K Coldwell C Partnering with health system operations leadership to develop a controlled implementation trial. *Implement Sci.* (2015) 11:22. 10.1186/s13012-016-0385-7 26912342 PMC4765154

[30] Israel B Schulz A Parker E Becker A. *Critical Issues in Developing and Following CBPR Principles. Community-Based Participatory Research for Health.* 3rd ed. San Francisco, CA: Jossey-Bass (2018). p. 31–46.

[31] Harvey N Holmes C. Nominal group technique: an effective method for obtaining group consensus. *Int J Nurs Pract.* (2012) 18:188–94. 10.1111/j.1440-172X.2012.02017.x 22435983

[32] Simoens S. Using the Delphi technique in economic evaluation: time to revisit the oracle? *J Clin Pharm Ther.* (2006) 31:519–22. 10.1111/j.1365-2710.2006.00780.x 17176357

[33] Cookson R Culyer A Norheim O. Principles of health equity. In: Cookson R Griffin S Culyer A Norheim O editors. *Distributional Cost-Effectiveness Analysis: Quantifying Health Equity Impacts and Trade-Offs.* Oxford: Oxford University Press (2021). p. 18–43. 10.1093/med/9780198838197.003.0002

[34] The White House. *Executive Order on Advancing Racial Equity and Support for Underserved Communities Through the Federal Government.* Washington, D.C: The White House (2021).

[35] Cookson R Griffin S Culyer A Norheim O. Designing a distributional cost-effectiveness analysis. In: Cookson R Griffin S Culyer A Norheim O editors. *Distributional Cost-Effectiveness Analysis: Quantifying Health Equity Impacts and Trade-Offs.* Oxford: Oxford University Press (2021). p. 44–68. 10.1093/med/9780198838197.003.0003

[36] Watkins J. Understanding value: the patients’ perspective. *Value Outcomes Spotlight.* (2022) 8:26–28.

[37] Jamieson M Govaart G Pownall M. Reflexivity in quantitative research: a rationale and beginner’s guide. *PsyArXiv*[Preprint]. (2022): 10.31234/osf.io/xvrhm

[38] Gunn C Bertelsen N Regeer B Schuitmaker-Warnaar T. Valuing patient engagement: Reflexive learning in evidence generation practices for health technology assessment. *Soc Sci Med.* (2021) 280:114048. 10.1016/j.socscimed.2021.114048 34052699

[39] National Council on Disability. *Quality-Adjusted Life Years and the Devaluation of Life with Disability: Part of the Bioethics and Disability Series.* (2019). Available online at: https://ncd.gov/sites/default/files/NCD_Quality_Adjusted_Life_Report_508.pdf (accessed May 10, 2022).

[40] Pyne J Tripathi S French M McCollister K Rapp R Booth B. Longitudinal association of preference-weighted health-related quality of life measures and substance use disorder outcomes. *Addiction.* (2011) 106:507–15. 10.1111/j.1360-0443.2010.03299.x 21205046 PMC3076048

[41] Papaioannou D Brazier J Parry G. How valid and responsive are generic health status measures, such as EQ-5D and SF-36, in schizophrenia? A systematic review. *Value Health.* (2011) 14:907–20. 10.1016/j.jval.2011.04.006 21914513 PMC3179985

[42] Mulhern B Mukuria C Barkham M Knapp M Byford S Brazier J. Using generic preference-based measures in mental health: psychometric validity of the EQ-5D and SF-6D. *Br J Psychiatry.* (2014) 205:236–43. 10.1192/bjp.bp.112.122283 24855127

[43] Neil A Carr V Mackinnon A Foley D Morgan V. Health-related quality of life in people living with psychotic illness and factors associated with its variation. *Value Health.* (2018) 21:1002–9. 10.1016/j.jval.2018.02.012 30098664

[44] Basu A Meltzer D. Implications of spillover effects within the family for medical cost-effectiveness analysis. *J Health Econ.* (2005) 24:751–73. 10.1016/j.jhealeco.2004.12.002 15960995

[45] Basu A. Economics of individualization in comparative effectiveness research and a basis for a patient-centered health care. *J Health Econ.* (2011) 30:549–59. 10.1016/j.jhealeco.2011.03.004 21601299 PMC3110511

[46] Patel U Blackmore M Stein D Carleton K Chung H. Costs and utilization for low income minority patients with depression in a collaborative care model implemented in a community-based academic health system. *Health Serv Res.* (2020) 55:106–7. 10.1111/1475-6773.13482

[47] Leung L Rubenstein L Post E Trivedi R Hamilton A Yoon J Association of veterans affairs primary care mental health integration with care access among men and women veterans. *JAMA Netw Open.* (2020) 3:e2020955. 10.1001/jamanetworkopen.2020.20955 33079197 PMC7576407

[48] Leung L Rose D Rubenstein L Guo R Dresselhaus T Stockdale S. Does mental health care integration affect primary care clinician burnout? Results from a longitudinal veterans affairs survey. *J Gen Intern Med.* (2020) 35:3620–6. 10.1007/s11606-020-06203-4 32948952 PMC7728981

